# Whole Genome Sequencing of Giant Schnauzer Dogs with Progressive Retinal Atrophy Establishes *NECAP1* as a Novel Candidate Gene for Retinal Degeneration

**DOI:** 10.3390/genes10050385

**Published:** 2019-05-21

**Authors:** Rebekkah J. Hitti, James A. C. Oliver, Ellen C. Schofield, Anina Bauer, Maria Kaukonen, Oliver P. Forman, Tosso Leeb, Hannes Lohi, Louise M. Burmeister, David Sargan, Cathryn S. Mellersh

**Affiliations:** 1 Kennel Club Genetics Centre, Animal Health Trust, Lanwades Park, Newmarket, Suffolk CB8 7UU, UK; jo@dwr.co.uk (J.A.C.O.); Ellen.Schofield@aht.org.uk (E.C.S.); Louise.Burmeister@aht.org.uk (L.M.B.); cathryn.mellersh@aht.org.uk (C.S.M.); 2 Department of Veterinary Medicine, University of Cambridge, Cambridge CB3 0ES, UK; drs20@cam.ac.uk; 3 Institute of Genetics, University of Bern, 3001 Bern, Switzerland; anina.bauer@vetsuisse.unibe.ch (A.B.); tosso.leeb@vetsuisse.unibe.ch (T.L.); 4 Department of Veterinary Biosciences, University of Helsinki, 00014 Helsinki, Finland; maria.kaukonen@helsinki.fi (M.K.); Hannes.Lohi@helsinki.fi (H.L.); 5 Department of Medical Genetics, University of Helsinki, 00014 Helsinki, Finland; 6 Folkhälsan Research Center, 00290 Helsinki, Finland; 7 Wisdom Health, Waltham-on-the-Wolds, Leicestershire LE14 4RS, UK; oliver.forman@effem.com

**Keywords:** canine, dog, progressive retinal atrophy, PRA, retinal degeneration

## Abstract

Canine progressive retinal atrophies (PRA) are genetically heterogeneous diseases characterized by retinal degeneration and subsequent blindness. PRAs are untreatable and affect multiple dog breeds, significantly impacting welfare. Three out of seven Giant Schnauzer (GS) littermates presented with PRA around four years of age. We sought to identify the causal variant to improve our understanding of the aetiology of this form of PRA and to enable development of a DNA test. Whole genome sequencing of two PRA-affected full-siblings and both unaffected parents was performed. Variants were filtered based on those segregating appropriately for an autosomal recessive disorder and predicted to be deleterious. Successive filtering against 568 canine genomes identified a single nucleotide variant in the gene encoding NECAP endocytosis associated 1 (*NECAP1*): c.544G>A (p.Gly182Arg). Five thousand one hundred and thirty canids of 175 breeds, 10 cross-breeds and 3 wolves were genotyped for c.544G>A. Only the three PRA-affected GS were homozygous (allele frequency in GS, excluding proband family = 0.015). In addition, we identified heterozygotes belonging to Spitz and Dachshund varieties, demonstrating c.544G>A segregates in other breeds of German origin. This study, in parallel with the known retinal expression and role of *NECAP1* in clathrin mediated endocytosis (CME) in synapses, presents *NECAP1* as a novel candidate gene for retinal degeneration in dogs and other species.

## 1. Introduction

Inherited retinal degenerations are a group of heterogeneous diseases characterized by loss of rod and/or cone photoreceptors eventually leading to vision loss. In humans, retinitis pigmentosa (RP) is the most common form, affecting 1 in 4000 worldwide [1] before middle age. Typically, night blindness is the first sign of RP caused by an initial depletion in rod photoreceptors followed by cones leading to a gradual reduction in vision.

Canine retinal degenerations present similarly to human forms of the disease and can be broadly categorized as stationary or progressive retinal disorders [2]. Collectively, these progressive forms are termed canine progressive retinal atrophies (PRA), with differences discussed in depth by others [3,4]. Variability in age of onset, aetiology, and rate of progression is observed in canine PRA, yet most forms share a similar ophthalmoscopic appearance. Fundus changes observed are bilateral and include tapetal hyper-reflectivity caused by retinal thinning, retinal blood vessel attenuation, and, in advanced stages, optic nerve head atrophy.

Canine retinal degeneration affects multiple breeds and is clinically and genetically heterogeneous across and within breeds. To date, 32 mutations have been associated with stationary and progressive forms across over 100 breeds. Twenty-five of these are associated specifically with PRA [5,6,7,8,9,10,11,12,13,14,15,16,17,18,19,20,21,22,23,24,25,26,27]. Although the majority of these are single gene autosomal recessive disorders, dominant [8] and X-linked forms [9,23] have been exemplified, as well as one age-modifying mutation [24]. Whilst there are PRA mutations that are shared by multiple breeds [11,19,28], many are private to a single breed [6,7,17,21,25] or are found in breeds sharing similar ancestral backgrounds, e.g., rod-cone degeneration-4 (RCD4) in Gordon and Irish Setters [19]. This is largely due to breed barriers and the existence of isolated populations within purebred dog breeds, where phenotypic variation within breeds is limited and dogs from the same breed are genetically more similar compared to those of different breeds [29]. In contrast, there have been PRA mutations found across seemingly unrelated breeds, including the RCD4 mutation present in Tibetan Terriers and the *PRCD* mutation found across many diverse breeds [11].

Currently, there is no treatment or cure for PRA; therefore, the use of genetic technologies to identify PRA-causing variants is crucial to facilitate diagnostic DNA test development for dog breeders and owners, with the aim to reduce the frequency of PRA-associated variants in dog breeds. Clinical eye screening schemes, including the British Veterinary Association/ Kennel Club/ International Sheep Dog Society (BVA/KC/ISDS) eye scheme in the UK (https://www.bva.co.uk/Canine-Health-Schemes/Eye-scheme/) and the ECVO (European College of Veterinary Ophthalmologists) Eye scheme (https://www.ecvo.org/hereditary-eye-diseases/eye-scheme), enable dog breeders and owners to screen for a list of inherited eye conditions in certain breeds to reduce the prevalence of certain eye diseases. Genetic testing complements clinical eye screening methods with the advantage of detecting known PRA mutations before breeding age or before clinical signs present. Similarities in eye size and clinical phenotypes of eye conditions in both dogs and humans presents the dog as an excellent model for studying inherited eye diseases in humans [30,31,32]. Studies of canine PRA offer a source for novel candidate gene identification and target gene discovery for retinal disease across species, including humans where a large proportion of patients still have an unknown molecular diagnosis.

Here, we describe a novel form of PRA implicating the gene encoding NECAP endocytosis associated 1 (*NECAP1*) in the Giant Schnauzer (GS) dog, a breed which originated in Germany in the 17th century. The GS is the largest of three sizes: Giant, Standard, and Miniature. Although the precise origins are unclear, it is thought the breed was developed using the Standard Schnauzer, Rottweiler, Great Dane, German Shepherd, and perhaps Bouvier De Flandres, although originally the GS was considered a rough-coated version of the German pinscher breeds [33]. The breed was imported to North America in the 1920s and 1930s, yet after the Second World War, the number of GS dogs diminished. Breeding stocks were introduced to the UK in the 1960s. Today, the three breed sizes do not interbreed and are recognized as distinct breeds. Currently, the GS is recognized by the BVA/KC/ISDS eye scheme as being affected with inherited cataracts but not PRA. To our knowledge, these affected GS dogs are the first PRA cases in the UK, and this is also the first instance that *NECAP1* has been implicated in retinal degeneration in any species. Although the small number of cases suggests this is a newly emerging form of PRA in the breed, identification of the genetic cause will enable further screening in the breed to confirm or exclude this hypothesis and improve our understanding of the aetiology of this form of PRA. The availability of a DNA test based on this variant will also prevent this form of PRA from becoming widespread in the GS population.

## 2. Materials and Methods

### 2.1. PRA Diagnosis and Sample Collection

**Figure 1:**
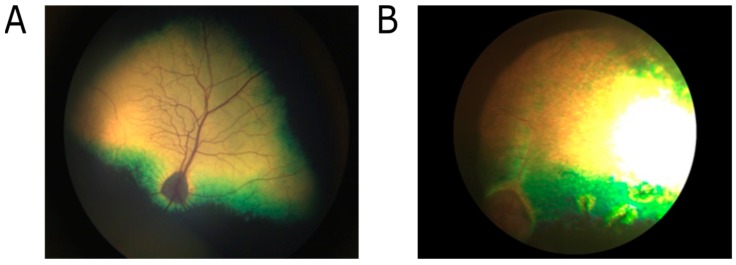
Fundus images captured showing (**A**) unaffected Giant Schnauzer (GS) dam at 11 years; (**B**) progressive retinal atrophies (PRA)-affected GS dog at 5 years of age. Observations of PRA cases include bilateral tapetal hyperrelectivity and retinal vascular attenuation.

PRA was initially diagnosed in a four-year-old GS dog by a board-certified veterinary ophthalmologist also qualified as a BVA/KC/ISDS eye scheme panelist (https://www.bva.co.uk/Canine-Health-Schemes/Eye-scheme/) and a diplomate of the European College of Veterinary Ophthalmologists at the Animal Health Trust. Ophthalmoscopic examination detected widespread tapetal hyper-reflectivity and retinal vascular attenuation, consistent with PRA (Figure 1). Follow-up examination of littermates detected a total of three of the seven dogs to be affected by PRA by board-certified veterinary ophthalmologists. The sire and dam were clinically unaffected at 8 and 11 years of age, respectively, and, therefore, considered likely to be obligate carriers of an autosomal recessive mutation causing PRA in this family. Neurological examination was performed in all dogs and revealed no abnormalities. A review of the medical records of each dog showed no history of neurological disease in any dog.

Collection of DNA samples from animals using buccal mucosal swabs was approved by the AHT Ethics Committee (ref no. 24-2018E). PRA-affected dogs (cases) were defined as presenting with clinical signs consistent with PRA and unaffected dogs (controls) as dogs clear of inherited eye diseases upon ophthalmoscopic examination. DNA was extracted from buccal mucosal swabs using the QIAamp DNA Blood Mini or Midi Kits (Qiagen, Manchester, UK). DNA concentration and purity were determined using the NanoDrop 1000 spectrophotometer (Thermo Fisher Scientific, Loughborough, UK) and/or the Qubit Fluorometer with the Qubit dsDNA broad range (BR) Assay Kit (Invitrogen, Loughborough, UK). Where possible, DNA samples with concentrations <10 ng/μL were concentrated using MultiScreen-PCR96 filter plates (Millipore, Watford, UK) or Microcon −30 kDa centrifugal filter units with ultracel-30 membrane (Millipore, Watford, UK). For the Finnish cohort, eye examinations were conducted by veterinary ophthalmologists board-certified by the European College of veterinary ophthalmologists; 3 mL EDTA-blood was collected under the permission of animal ethical committee of County Administrative Board of Southern Finland (ESAVI/343/04.10.07/2016) and DNA was extracted from white blood cells using a semi-automated Chemigen extraction robot (PerkinElmer Cheagen Technologie GmbH, Baeswieler, Germany) according to manufacturer’s instructions. Breed-specific allele frequencies for the *NECAP1* variant were determined using cohorts of dogs genotyped by the Animal Health Trust, UK; University of Bern, Switzerland; and University of Helsinki, Finland. A sample cohort genotyped by Mars Veterinary, UK, provided further screening of the *NECAP1* variant across multiple dog breeds, including mixed breed dogs and dogs of unknown breeds.

### 2.2. Exclusion of Known Retinal Mutations

Twenty-six previously published retinal mutations (Supplementary Table S1) were screened for using a genotyping-by-sequencing method. Primer pairs were designed spanning each mutation to amplify products between 110–130 bp in size. Fifteen primer pairs were pooled and a multiplex PCR was performed. Multiplex PCR and thermal cycling conditions are listed in Supplementary Tables S2 and S3. Purification was carried out after each thermal cycling reaction using AMPure XP beads (Beckman Coulter, High Wycombe, UK), according to manufacturer’s instructions, and using 1:1.75 ratio of beads to DNA-containing solution. Adaptor ligation was performed followed by amplification to create sequencing libraries. Five microliters (μL) of each sample library was pooled and quantified using KAPA library quantification kit, according to manufacturer’s instructions (Kapa Biosystems, Wilmington, MA, USA). The final library was diluted to 15 picomoles (pM) and loaded into a 150 bp v3 kit cartridge (Illumina, Cambridge, UK) for single ended sequencing on the MiSeq sequencing platform (Illumina). FASTQ files were aligned to the canine genome assembly CanFam3.1 (Sep.2011. Broad CanFam3.1/canFam3, Ensembl (www.ensembl.org; Dog release 89) [34]) using BWA, producing BAM files. BAM files were visualized in Integrative Genomics Viewer (IGV) [35,36]. Large insertions or deletions or variants within repetitive regions, applicable for a total of eight mutations, were genotyped using PCR amplification, followed by amplified fragment analysis or visualization on an agarose gel using gel electrophoresis.

### 2.3. Whole Genome Sequencing

The four GS samples selected for whole genome sequencing (WGS) (2 PRA cases and both unaffected parents; study accession number PRJEB32096) were normalized to 25 nanograms per μL (ng/μL), and 1000 ng was sent for sequencing, outsourced to Edinburgh Genomics, UK. Illumina sequencing of a TruSeq Nano library on a HiSeq X sequencing platform generated a dataset of approximately 30× coverage of the dog genome. Reads were aligned to the canine reference genome (CanFam3.1) using BWA-mem, variant calls were made using GATK (HaplotypeCaller) and base quality score recalibration, and indel realignment and duplicate removal were performed [37]. Single nucleotide polymorphism (SNP)and small insertion/deletion (INDEL) discovery was performed using standard hard filtering parameters or variant quality score recalibration according to GATK Best Practices recommendations [38,39]. Sequencing reads and variants were visualized in IGV. Genomic Variant Call Format (VCF) files from 116 canine WGS were combined by HaplotypeCaller into a multi-sample VCF file. Variant Effect Predictor (VEP) was run on the merged VCF file for cross-genome analysis.

### 2.4. Variant Filtering

Variants from WGS data were filtered appropriately for an autosomal recessive mode of inheritance, i.e., variants were retained if cases were homozygous, parents heterozygous, and controls homozygous for the alternate allele. An in-house analysis pipeline generated an effect score for each variant, depending on its predicted effect on the protein sequence and whether it is predicted to be deleterious. Variant filtering retained variants with a high effect score, including those resulting in premature start/stop codons, splice site variants, nonsense and missense variants, frameshift variants, and in-frame deletions. Study co-authors (C.S.M., O.P.F., H.L., T.L.) are members of the Dog Biomedical Variant Database consortium (DBVDC), which contained 452 WGS at the time of the study; these sequences were used for additional variant filtering. The Ensembl genome browser (www.ensembl.org; Dog release 89) [34] and UCSC genome browser (http://genome.ucsc.edu/) were used to obtain canine genome sequence (Sep.2011. Broad CanFam3.1/canFam3) to interrogate regions. Mutation Analyzer, PolyPhen-2, Mutation Taster2, and SIFT (sorting intolerant from tolerant) were used to predict the impact of amino acid substitutions on the structure and function of proteins [40,41,42,43].

### 2.5. Variant Screening

Primers and allelic discrimination probes were designed using Primer3 [44,45] and obtained from Integrated DNA Technologies (IDT, Leuven, Belgium) (Supplementary Table S4). Probes were PrimeTime ZEN double-quenched qPCR probes containing a 5’ fluorophore, 3’ Iowa Black® FQ (IBFQ) quencher and proprietary, internal ZEN™ quencher. A 5′ HEX™ fluorophore was used to determine the reference allele and a FAM™ fluorophore to label the alternate allele. Individual PrimeTime assays were re-suspended in ultrapure water to a 40× mix and combined. Allelic discrimination assays were carried out using Luna Universal qPCR Master Mix (New England Biolabs, Hertfordshire, UK) on a StepOnePlus™ Real-Time PCR system (Thermo Fisher Scientific, Loughborough, UK) using the following cycling conditions: 25 °C for 30 s; 95 °C for 3 min; 35 cycles of 95 °C for 3 s, followed by 60 °C for 10 s; and a final post-PCR stage at 25 °C for 30 s. Results were analyzed using Applied Biosystems StepOne Software v2.3. For the Finnish cohort, primers were designed using Primer3 (forward: 5’-TGGTTTCTTTCCCCTACTCCC; reverse: AATGGGTGGTGGAGTGACAT) to amplify DNA by PCR using the following cycling conditions: initial denaturation at 95 °C for 5 min; 37 cycles denaturing at 95 °C for 30 s, annealing at 59 °C for 30 s, and elongation at 72 °C for 45 s; followed by a final elongation step at 72 °C for 10 min. PCR components are listed in Supplementary Table S5 and PCR products were sequenced by Sanger sequencing.

### 2.6. Sanger Sequencing of NECAP1 Variant

To confirm the c.544G>A (p.Gly182Arg) SNV and its segregation in all three GS PRA cases, PCR followed by Sanger sequencing were performed. Forward (5’–GGCTTCAAGGAAGGACAGACT-3’) and reverse (5’- GATACGATGATTCCTCAAAGTTAAA-3’) primers were designed using Primer3 to amplify a 350 bp product flanking the candidate variant using the following PCR conditions: denaturation at 95 °C for 5 min; 35 cycles at 95 °C for 30 s, 58 °C for 30 s, and 72 °C for 30 °C followed by a final elongation step at 72 °C for 5 min. PCR products were purified on a MultiScreen-PCR96 filter plate (Millipore, Watford, UK) and sequenced using the Sanger method using Bigdye v3.1 chemistry (Life Technologies Ltd., Loughborough, UK) and the following conditions: 96 °C for 30 s; 44 cycles at 92 °C for 4 s, 55 °C for 4 s, and 60 °C for 1.5 min. Isopropanol precipitation of sequencing reaction products removed excess reagents and precipitated DNA was resuspended in 10 µL Hi-Di Formamide (Applied Biosystems, Loughborough, UK). Sequencing products were separated on an Applied Biosystems (ABI) 3130xl genetic analyzer and data analyzed using the Staden software package (http://staden.sourceforge.net/).

### 2.7. Expression of NECAP1 in Canine Retina, Using RNAseq Data

RNAseq data generated from retinal RNA of a Petit Basset Griffon Vendeen (PBGV) control dog for a previous unrelated project [46] was used to determine gene expression. Three genes known to be implicated in retinal degeneration and expressed in retinal tissue (*RPGRIP1*, *RHO*, and *GNAT1*) and three genes with low/no expression in human retinal tissue (*ANXA1*, *MYH41*, *MYOT*) were selected as control genes based on RNAseq expression data published in GeneCards [47]. The total number of reads aligned to each of the six control genes was obtained using BEDTools v2.17.0 “coverage” tool [48] and compared to that of *NECAP1*. An average number of reads per exon was calculated for each gene.

### 2.8. Haplotype Analyses

A total of eight SNVs, four either side of the *NECAP1* variant, that were homozygous in the cases and heterozygous in the parents were selected for genotyping. SNVs were situated up to 573 kilobases (kb) downstream of the variant and 301 kb upstream. Primers were designed using Primer3 and products amplified using PCR conditions, purification, and Sanger sequencing, as described above. Primers are listed in Supplementary Table S6. SNVs surrounding the variant were genotyped across two GS PRA cases, both GS parents, one control GS, one heterozygous Giant Spitz, six heterozygous Miniature Longhaired Dachshunds, and a control Miniature Longhaired Dachshund to determine a disease-associated haplotype.

### 2.9. Autozygosity Mapping

Illumina Canine HD array SNP positions were extracted from the four GS WGS data into binary files using PLINK2 (www.cog-genomics.org/plink/2.0/) [49] and an in-house PERL script. PLINK v1.07 [50] was used to identify runs of homozygosity (ROH) using sliding windows across the SNP data using the default values: -homozyg-window-kb 5000 (length in kb of the sliding window); -homozyg-window-snp 50 (minimum number of SNPs that the sliding window requires); -homozyg-window-het 1 (allowing 1 heterozygous SNP in a window to tolerate genotyping errors); -homozyg-window-missing 5 (allowing up to 5 missing calls in a window); -homozyg-window-threshold 0.05 (proportion of overlapping windows required as homozygous to define a given SNP in a ROH); -homozyg-snp 100 (sliding window of 100 SNPs); -homozyg-kb 1000 (sliding window of 1000 kb/ 1 megabase (Mb)); -homozyg-density 50 (minimum density of 1 SNP per 50 kb); and -homozyg-gap 1000 (split a segment into two if the distance between two SNPs is >1000 kb, i.e., retaining ROH >1 Mb in size). ROH overlapping with one or both parents were removed leaving those homozygous only in the cases, leaving, by definition, ROH heterozygous in the parents. Genes within each ROH block were cross-referenced with the RetNet database [51] and RetNet gene regions were visualized in the GS WGS data for high impact SNVs or structural variants.

## 3. Results

### 3.1. Exclusion of Known Retinal Mutations

Screening for 26 previously published retinal mutations (Supplementary Table S1) revealed all PRA-affected GS were homozygous for the wildtype allele for all these mutations.

### 3.2. NECAP1 Variant Identification

**Figure 2:**
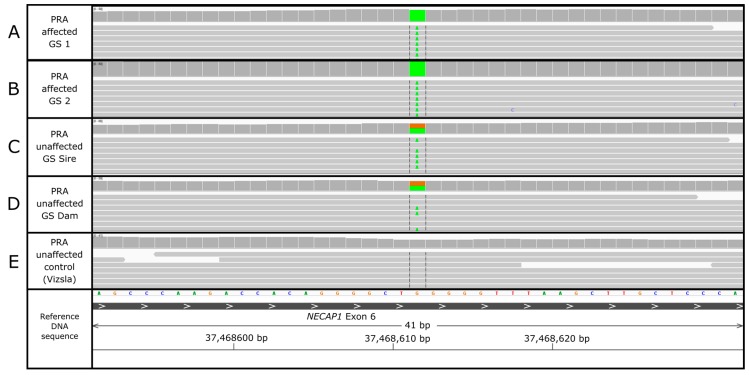
Whole genome sequencing (WGS) analysis in Integrative Genomics Viewer (IGV) of GS PRA cases homozygous (**A**,**B**) for the c.544G>A (p.Gly182Arg) single nucleotide variant (SNV) in exon 6 of 8 of NECAP endocytosis associated 1 (*NECAP1*), unaffected sire (**C**) and dam (**D**) heterozygous for the variant, and Vizsla control homozygous for the wildtype allele (**E**).

Genome sequencing data of two PRA-affected full-siblings were compared with corresponding data of their unaffected parents and 112 control dogs of various breeds. Variants were filtered by the predicted effect of each variant on the transcript and protein and then based on those segregating appropriately for an autosomal recessive disorder. A total of 25,658,899 variants were detected amongst 116 canine genomes of multiple breeds, including the four GS genomes. Filtering for protein changing variants reduced this number to 102,716 variants. Five of these high effect variants segregated appropriately in both cases and parents and were absent from our initial control set of 112 canine genomes. Additional filtering against genomes in the DBVDC (452 genomes of 94 individual breeds, eight cross breeds, and three wolves) further reduced this set of five variants down to three. Two variants were situated within a novel gene, ENSCAFG00000030684, for which no human homologues exist nor do any annotated genes lie nearby. This left a single candidate missense variant in the gene *NECAP1* encoding NECAP endocytosis associated 1 for which, aside from the two GS parents, only one additional heterozygous dog (Giant Spitz breed) was identified in the full dataset of 568 genomes (WGS controls and DBVDC cohort). The c.544G>A (p.Gly182Arg) SNV is in exon 6 of 8 in *NECAP1* (chr27:37,468,611) (CanFam 3.1 assembly; Figure 2) and is predicted to cause a glycine to arginine amino acid substitution. This glycine is highly conserved across 31 mammals, including humans and mice at this position.

To assess whether the c.544G>A variant amino acid substitution affects protein function, bioinformatics tools Mutation Analyzer, PolyPhen-2, Mutation Taster2, and SIFT were used to predict the effect of the missense variant. Mutation Analyzer and PolyPhen-2 predicted the amino acid change to be rarely tolerated and probably damaging to the protein, respectively. Mutation Taster predicted the amino acid substitution to be disease-causing and SIFT predicted the change to be deleterious with a SIFT score of 0.03.

### 3.3. Variant Screening

**Figure 3:**
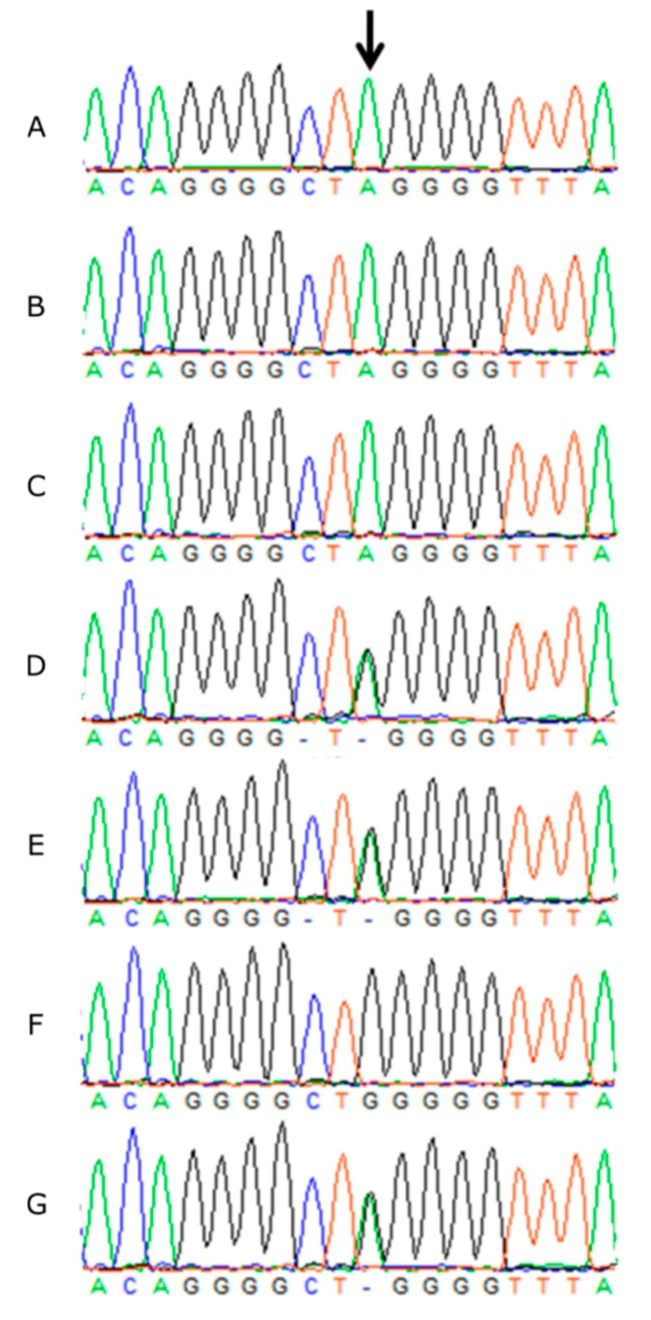
Sanger sequencing traces showing the c.544G>A SNV in *NECAP1* in three PRA affected GS DNA (**A**–**C**); the heterozygous unaffected sire (**D**) and dam (**E**); a homozygous wildtype unaffected sibling (**F**), and a heterozygous unaffected sibling (**G**).

Sanger sequencing confirmed all three PRA-affected GS dogs were homozygous for the mutant allele (A/A), both obligate carrier parents were heterozygous (G/A) and the remaining four unaffected littermates were either heterozygous or homozygous for the wildtype allele (G/G) (Figure 3).

To investigate whether the variant is common in the breed, additional GS dogs were genotyped for the c.544G>A variant. Six additional GS heterozygotes were initially found. Screening an additional 427 dogs of 122 breeds, including 65 Miniature Schnauzers, identified one Miniature Longhaired Dachshund that was heterozygous for the c.544G>A variant.

### 3.4. Population Screening

The presence of the *NECAP1* variant in additional breeds led to screening of Miniature Longhaired Dachshunds and Spitz varieties. In total, the candidate variant was genotyped in 5130 dogs of 175 breeds, 10 cross breed dogs, and 3 wolves, including a total of 322 GS dogs, were genotyped for c.544G>A (p.Gly182Arg). Only the three PRA-affected GSs were homozygous for the variant and 18 heterozygotes were identified, nine of which were closely related (up to three generations) to the probands, and nine were either known to be not closely related to the affected dogs based on pedigree information, or deemed unknown due to lack of pedigree information. Allele frequency in GS dogs, excluding closely related dogs, was 0.015. Genotyping cohorts of additional breeds of German origin, including Spitz and Dachshund varieties, identified heterozygotes of the variant. Genotyping data were compiled and allele frequencies determined (Table 1). Four PRA affected dogs (two Miniature (Klein) Spitz and two Pomeranian) were included in the testing cohorts: these were homozygous wildtype suggesting that in these two breeds there are multiple forms of PRA. Out of the total 5130 canids genotyped for the *NECAP1* variant, the only dogs homozygous for the variant were the three GS PRA cases.

One of the study cohorts (Mars Veterinary, UK) consisted of a combination of unconfirmed breeds and mixed breed dogs (*n* = 3162) in which 21 additional heterozygotes were identified. Heterozygotes of the following purebreds were identified: Miniature Longhaired Dachshund (*n* = 2), Shorthaired Dachshund (*n* = 1), Miniature Spitz (*n* = 2), and GS (*n* = 1); 13 mixed breed dogs were identified for which breed compositions could be estimated (Supplementary Table S7). Breed type could not be estimated for two heterozygotes in this cohort. As this cohort included various mixed breed or unknown breeds of dogs, these were excluded from the breed specific allele frequency calculations.

### 3.5. NECAP1 Is Expressed in Canine Retina

**Figure 4:**
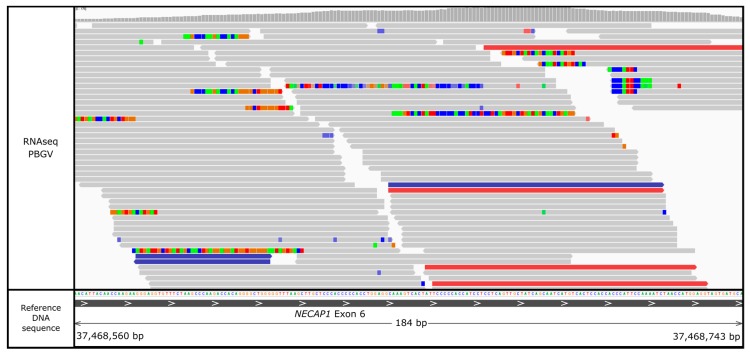
Alignment of RNAseq data in IGV from a control Petit Basset Griffon Vendeen (PBGV) retina to the CanFam3.1 canine genome show reads mapping to *NECAP1*.

A qualitative measure of expression was observed by extracting the total number of reads per exon from PBGV retinal RNAseq data and calculating a mean number of reads per exon for each gene (Table 2). A total of 749 reads aligned over all eight exons of *NECAP1*, with 288 reads aligned specifically over exon six (Figure 4), providing evidence that *NECAP1* is expressed in canine retinal tissue.

### 3.6. Haplotype Analyses

To establish whether the *NECAP1* variant is due to recurrent independent mutation events or inherited identically by descent, SNVs flanking the variant were genotyped in the two GS PRA cases, both GS parents, one control GS, one heterozygous Giant Spitz, six heterozygous Miniature Longhaired Dachshunds, and a control Miniature Longhaired Dachshund. SNVs present as homozygous for the alternate allele in the GS PRA cases and heterozygous in the obligate carrier parents were determined. Shorter haplotypes were revealed in the Giant Spitz and Miniature Longhaired Dachshund, with the shortest in the Dachshund, compared to that of the GS, suggesting the shared region may be an ancestral and ancient haplotype (Figure S1).

### 3.7. Autozygosity Mapping

**Figure 5:**
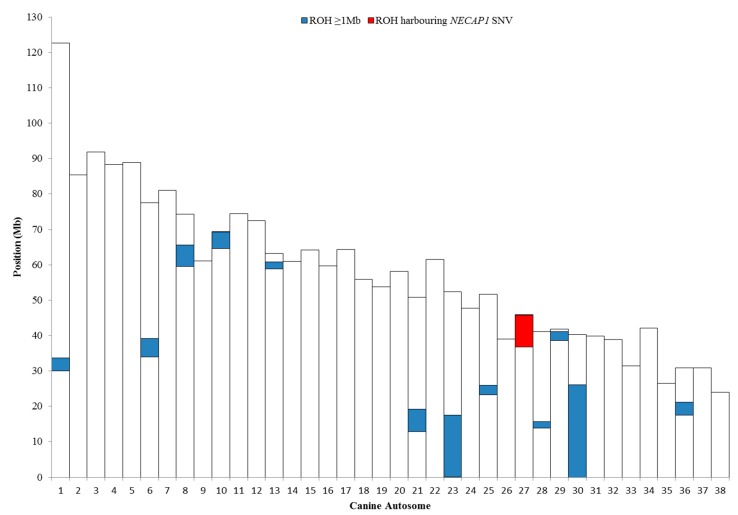
Chromosomal positions of runs of homozygosity (ROH) greater than 1 Mb in size (blue), on canine autosomes in the two GS PRA cases, as obtained through PLINK analysis. The red block indicates the 9.03 Mb ROH on chromosome 27 in which the *NECAP1* variant lies.

After filtering and quality control, 106,298 SNPs were tested through PLINK and identified thirteen ROH that were over 1 Mb in size and shared exclusively by both GS PRA cases (Table 3, Figure 5).

Eight ROH harbored at least one RetNet gene; however, no structural variants nor protein-changing variants were detected upon visualization of WGS data from both PRA-affected cases and unaffected parents. The *NECAP1* variant lies within a 9.03 Mb ROH on canine chromosome 27 (CANFA27).

## 4. Discussion

In this study, we presented novel findings associated with, to our knowledge, a novel form of PRA in the GS. The use of WGS technologies has thrived in the past five years and is being increasingly used in genetic studies to identify causative mutations associated with inherited diseases across species. This has led to success in identifying mutations in canine inherited diseases using a very small number of cases and is a cost-effective approach when genome-wide association study (GWAS) or other positional approaches are unattainable with small sample numbers. Through WGS a quartet of GS dogs (two full-siblings and both parents), we have identified a novel candidate missense variant in *NECAP1*; this gene has not formerly been implicated in retinal degenerations in any species. By sequencing two cases and their unaffected parents, comparing the WGS of this quartet with those of a large bank of dogs of other breeds, and assuming an autosomal mode of inheritance, our ability to filter candidate variants was considerable.

The *NECAP1* gene on CANFA27 is comprised of eight exons spanning 46 kb. *NECAP1* encodes NECAP endocytosis associated 1, also termed adaptin ear binding coat associated protein 1, a subunit of the adaptor protein-2 (AP-2) complex involved with clathrin mediated endocytosis (CME) in synapses [52]: a vesicular transport event that primarily initiates the entry of clathrin-coated vesicles (CCVs) into cells. The process involves nutrient uptake, signalling and recycling of receptors, as well as playing a role in synaptic vesicle reformation [53,54] which is crucial for normal cell function. AP-2 is central to CME, during which a subunit of AP-2 binds to the clathrin coat membrane and traps various transmembrane proteins, including cargo receptors, to enrich CCVs [52]. CME is present, albeit playing a minor role, in retinal ribbon synapses in photoreceptors and bipolar cells of the retina.

A nonsense mutation in *NECAP1* has previously been associated with a recessive early infantile epileptic encephalopathy (EIEE) in humans [55]. Clinical signs of EIEE include severe intractable seizures from early infancy with a progressive change in frequency and intensity. No retinal abnormalities were detected in the study by Alazami et al. [55] following electroretinogram of patients homozygous for the nonsense variant. However, patients with other forms of EIEE have been reported to show signs of retinal degeneration in addition to typical EIEE clinical signs [56,57]. One study described an infant that was homozygous for a missense variant in the *ARV1* gene that presented with a form of autosomal recessive EIEE. Ophthalmoscopic evaluation revealed minimal pupillary response to light leading to a diagnosis of retinal degeneration. The patient died at one year of age [56]. A second study identified a novel single base pair deletion in *GNB5* causing a syndromic form of EIEE. Patients presented with signs of EIEE, as well as retinal degeneration, cardiac abnormalities, severe neurological developmental delay, and premature sudden death [57]. Although these three discussed forms of EIEE are caused by mutations in different genes, it can be speculated that the patient that was homozygous for the *NECAP1* variant [55] that died around seven years of age, may not have survived long enough to develop retinal changes. Arguably, patients reported by in Palmer et al. [56] and Turkdogan et al. [57] may have had both EIEE and retinal degeneration as a result of genetically distinct diseases. Although no MRI scans nor neurological examinations were carried out on the GS dogs in our study, no obvious neurological abnormalities were noted by the veterinary ophthalmologist(s) or by their owner. It can also be hypothesized that the predicted premature truncation of the NECAP1 protein by the nonsense mutation described by Alazami et al. [55] has a more extreme consequence on the protein than a missense SNV and therefore could account for the severity of the disease in humans compared with our GS cases. Currently, there are no reports of *NECAP1* implicated in retinal degeneration in any species.

While *NECAP1* was initially reported to be primarily expressed in brain tissue [52], it has also been detected throughout the central nervous system in mice, including the spinal cord [55] and retina of mice and beagle dogs [58,59]. Retinal RNAseq data generated for an unrelated project from a PBGV dog revealed that *NECAP1* is highly expressed in canine retinal tissue. Previous work involving RP mouse models demonstrated the role of CME in retinal cells. Park et al. showed that CME is present in the rod bipolar cell axon terminals in the mouse retina, suggesting that this process may be important for normal synaptic function in the mammalian retina. RP mouse models of early onset retinal degeneration produced by a mutation in the *Pde6b* gene showed changes in the postsynaptic cells of the rod and cone photoreceptors in the retina. Specifically, bipolar cells in rod photoreceptor cells, involved in the rod neural pathway, generate ribbon synapses that are usually formed by postnatal day 14 in mice retinas [60]. Preferential changes in endocytosis at the rod bipolar ribbon synapses were reported in mice exhibiting rapid photoreceptor degeneration, including abnormal synaptic ribbons in rod bipolar cells where clathrin was no longer expressed in comparison to a control mouse retina [60]. Moreover, an example of a gene involved in CME causing RP in humans is the receptor expression enhancing protein-6 (*REEP6*) gene [1,61]. *REEP6* is involved in intracellular trafficking of CCVs to membrane sites, and a loss of *REEP6* function resulted in photoreceptor cell death in mouse models [61]. Studies using Drosophila as an RP disease model highlight the significance for endocytosis of rhodopsin, a light sensitive protein which is crucial component of the phototransduction cascade triggering vision. Endocytosis of metarhodopsin, the active form of rhodopsin, in rhabdomeres present in Drosophila photoreceptors, is essential for photoreceptor maintenance. Endocytosis of metarhodopsin is facilitated by the binding of arrestin to AP-2, hence when this pathway is compromised, metarhodopsin accumulates in the rhabdomeres and leads to the degeneration of photoreceptors [62]. Examples of rhodopsin accumulation caused by mutations in genes involved in endolysosomal pathways [63,64] suggest reduced endocytosis in photoreceptors lead to an accumulation of rhodopsin in photoreceptor cells, leading to photoreceptor cell death and ultimately retinal degeneration.

Although there is no direct functional evidence supporting the involvement of *NECAP1* on retinal function, mutations discussed in endolysomal trafficking genes with similar molecular mechanisms to *NECAP1* suggest this is a provocative novel candidate gene for retinal degeneration. Studies discussed in mice suggest that CME is essential for processes at the rod bipolar ribbon synapses in the mammalian retina [60,61], therefore, we can venture that if the expression or function of a gene regulating this process is disrupted, its role in maintaining rod bipolar ribbon synapses and subsequently photoreceptor function may be compromised. We speculate that the NECAP1 glycine to arginine substitution identified in GS dogs with PRA impacts protein function, and that potential inactivation of the AP-2 adaptor complex could disrupt endocytosis in retinal neurons, such as ribbon synapses in photoreceptor and bipolar cells. We hypothesize that, as a consequence of CME disturbance in the retina, rhodopsin accumulates in the photoreceptors, leading to cell death and retinal degeneration. Despite the addition of verifying *NECAP1* expression in a normal dog retina, a notable limitation of our study is the absence of tissue from affected animals, which prevented any histology, gene expression or protein analysis to be undertaken. Although PRA is a blinding disease, it rarely requires enucleation on welfare grounds and affected dogs are typically lost to follow up. From a biological research perspective, this limits RNA analysis when novel candidate genes are implicated. Our study presents further possibilities to explore *NECAP1* involvement in canine and human retinal degenerations.

We have shown the *NECAP1* missense variant segregates with disease in the studied GS family, as well as being detected in heterozygous state in additional breeds. Our screening cohort included 65 Miniature Schnauzers, in which no copies of the *NECAP1* variant were identified. This is not too extraordinary, as the Miniature Schnauzer and GS are recognized as distinct breeds. Additional genotyping of Standard Schnauzers and other breeds used to develop the GS breed would be more informative; however, sample numbers of these breeds in our DNA bank limited this scope. Due to the presence of our candidate variant on a shared identical haplotype in additional breeds, we hypothesize that it is due to an ancestral founder mutation event and inherited identically by descent. It is not uncommon for deleterious mutations to be shared across breeds which appear to be unrelated, for example the *PRCD* mutation has been detected in a various diverse dog breeds [11]. The German Spitz varieties, Dachshund varieties, and GS are all breeds seemingly of German ancestry: the GS breed, once known as the Munich Schnauzer, was developed in Germany using the Standard Schnauzer, Rottweiler, Great Dane, German Shepherd, and perhaps Bouvier De Flandres [33]. Both the Dachshund and those Spitz varieties carrying the *NECAP1* variant also originated in Germany. Shared haplotype analysis identifies only the Standard Schnauzer, Airedale Terrier, and Black Russian Terrier as ancestors to the GS, however, the study by Parker at al. examined haplotypes >232 kb, which are likely to detect only more recent events. The *NECAP1* haplotype we describe in the Miniature Longhaired Dachshund is only half this size and the Giant and Medium Spitz were not examined [65]. We hypothesize that the founding haplotype was present in a dog of German ancestry, most likely ancestors of the Dachshund varieties, and prior to the development of these other breeds. Typically, the lengths of common shared ancestral chromosomal segments in a population are short due to the occurrence of recombination events over time, where the shorter haplotype in the Dachshund indicates increased recombination on what once was a longer haplotype. The detected haplotype may have been common in the population when the *NECAP1* variant arose and, therefore, normal copies of this haplotype were also present.

We hypothesize that *NECAP1* is associated with retinal degeneration and is the cause of PRA in our GS study, despite the absence of any epileptic or neurological signs. The breed allele frequency of 0.015 suggests that this form of PRA in the GS is rare, resulting in insufficient cases to conduct a genome-wide association study. Due to the limited number of DNA samples from affected dogs, we opted to directly utilize a comprehensive WGS approach to identify candidate causative variants, with the advantage of sequencing both unaffected parents as obligate carriers to aid our analysis. Retrospective autozygosity mapping showed the *NECAP1* variant lies within a run of homozygosity in the cases only, indicating it is identical by descent and thus providing further evidence for its association with the disease. One limitation to this WGS approach is the detection of structural variants, such as inversions, transposons, or large insertions/deletions which our current WGS pipeline cannot currently identify using WGS alone, therefore, the presence of structural variation causing disease in this study cannot be excluded. Despite this, evidence discussed suggests *NECAP1* is a provocative candidate gene for retinal degeneration.

## 5. Conclusions

We have identified a novel candidate variant in *NECAP1* for PRA in the GS. We have demonstrated that this variant segregates in other German breeds, suggesting it may be due to an ancestral mutation event. To date, *NECAP1* has not been associated with retinal degeneration in any species. This study, in parallel with the known function and well-documented role of *NECAP1* in clathrin-mediated endocytosis in retinal cells, presents *NECAP1* as a novel candidate gene for retinal degeneration in dogs and other species.

## Figures and Tables

**Table 1:** Total allele frequency of *NECAP1* single nucleotide variant (SNV) in multiple breeds of dog.

Breed	*NECAP1*+/+	*NECAP1*−/+	*NECAP1*−/−	Allele Frequency
Giant Schnauzer (GS)	301	18	3	0.037
GS *	300	9	0	0.015
Giant Spitz	106	7	0	0.031
Medium Spitz	146	5	0	0.017
Miniature Spitz	106	8	0	0.035
Pomeranian Spitz	56	3	0	0.025
Miniature Longhaired Dachshund	157	8	0	0.024
175 other breeds or unknown breeds	4206	0	0	

* excluding third generation relatives to probands from known pedigree information.

**Table 2:** The total number of exonic reads in three genes expressed in human retinal tissue and three genes with low/no expression in human retina was extracted and the mean number of reads per exon calculated. Comparison of these six control genes and the values generated for *NECAP1* using canine RNAseq data showed reads aligning to *NECAP1* providing evidence of gene expression in the Petit Basset Griffon Vendeen (PBGV) control canine retina.

Gene	CanFam3.1 Chromosomal Coordinates	Total Number of Exonic Reads	Mean Number of Reads Per Exon
*RPGRIP1*	15: 18316887-18387548	1458	58
*RHO*	20: 5632150-5637404	461640	92328
*GNAT1*	20: 39129469-39133156	118800	13200
*ANXA1*	1: 84744444-84763121	120	9
*MYH41*	5: 34748657-34767076	3	0
*MYOT*	11: 25591798-25592328	40	4
*NECAP1*	27: 37460607-37506942	749	94

**Table 3:** PLINK analysis identified 13 runs of homozygosity (ROH) exceeding 1 Mb in size present in both PRA affected GS dogs. The *NECAP1* single nucleotide variant (SNV) lies within the 9.03 Mb ROH identified on chromosome 27.

CanFam3.1 Chromosomal Coordinates	Size (Mb)
1:29970719-33682751	3.71
6:33954067-39111638	5.16
8:59486837-65640116	6.15
10:64657382-69250914	4.59
13:58887427-60841228	1.95
21:12842407-19235893	6.39
23:54039-17493091	17.44
25:23239981-26009032	2.77
27:36722004-45753342	9.03
28:13922125-15662789	1.74
29:38572915-41054487	2.48
30:1366-26126946	26.13
36:17518975-21096460	3.58

## Supplementary Materials

The following are available online at https://www.mdpi.com/2073-4425/10/5/385/s1, Supplementary Table S1: Twenty-six known canine retinal mutations were excluded as causal variants in the GS cohort, Supplementary Table S2: Multiplex PCR amplification using pooled primers, Supplementary Table S3: Thermal cycling conditions for multiplex PCR amplification using pooled primers, Supplementary Table S4: Primer and probe sequences for allelic discrimination assay to determine *NECAP1* genotypes, Supplementary Table S5: PCR amplification for the Finnish cohort, Supplementary Table S6: Primer sequences to amplify SNVs downstream and upstream *NECAP1* variant to determine a disease associated haplotype, Supplementary Table S7: Known breed compositions of 13/15 *NECAP1* heterozygous mixed breed dogs as tested by Wisdom Health, UK, Figure S1: To build a disease-associated haplotype, a total of eight SNVs up to 301 kb upstream and 573 kb downstream of the *NECAP1* variant were genotyped in two PRA affected GS *NECAP1* homozygotes, both NECAP1+/− unaffected parents, and an unaffected *NECAP1*+/+ GS; one *NECAP1*+/− Giant Spitz from the DBVDC; six *NECAP1*+/− Miniature Longhaired Dachshunds and one *NECAP1*+/+ Miniature Longhaired Dachshund. A disease associated haplotype (shaded orange) was determined. The alternate allele is shaded yellow.
