# Supplementary material for: Whole Genome Sequencing of Giant Schnauzer Dogs with Progressive Retinal Atrophy Establishes NECAP1 as a Novel Candidate Gene for Retinal Degeneration
Source: Genes (Basel). 2019 May 21;10(5):385. doi: 10.3390/genes10050385 (PMC6562617; doi:10.3390/genes10050385)
Supplement: Supplementary file 1 [file genes-10-00385-s001.pdf]

Supplementary Table 1. Twenty-six known canine retinal mutations were excluded as causal variants in the GS cohort.

Form of Retinal Degeneration	Locus or Abbreviation	Gene Involved	Primer Sequences	References
		Pooled for multiplex PCR		
Cone degeneration	CD	*CNGB3*	F-CCCACTTACCATTATGTCTCCTC; R-TCAAACACCAGACAACACACA	Sidjanin, Lowe [1], Yeh, Goldstein [2]
Canine achromatopsia	ACHM	*CNGA3*	SNP_F-AGTGGGCGTCCTAATCTTTG; SNP_R-CGGAATTGCATGTACTGCTT; del_F-CCTGATCGACGAGGACGTG; del_R-CGTTGTACTCGGCCAGGAG	Tanaka, Dutrow [3]
Congenital stationary night blindness/retinal dystrophy	CSNB	*RPE65*	F-GACAATGCCCTTGTTAACGTC; R-AGCATTTCGTCCTACCTGCTT	Aguirre, Baldwin [4], Veske, Nilsson [5]
Canine multifocal retinopathy	CMR1	*BEST1/ VMD2*	F-CGTCACCTACTCAAGCCAAGT; R-TAGCTGAGCAGGAAGATGAGG	Guziewicz, Zangerl [6]
Canine multifocal retinopathy	CMR2	*BEST1/ VMD2*	F-GGCCCTCCCCATCCACTG; R-CAGGGCACCCAGAACGTGT	Guziewicz, Zangerl [6]
Canine multifocal retinopathy	CMR3	*BEST1/ VMD2*	F-AAGGCCTGGGAGATTAAGGAG; R-CTGACTGTCCAGATGGGAAGA	Zangerl, Wickstrom [7]
Generalised PRA	gPRA	*CCDC66*	F-GCTCATACTGCTTAGATGTCTCTTG; R-CAATACTTCCAATCAGCCAAA	Lippmann, Jonkisz [8], Dekomien, Vollrath [9]
Progressive retinal atrophy	PRA	*CNGB1*	F-CCACCCAGGCTCAGCAG; R-TCAGGCAGCCCACCAAT	Ahonen, Arumilli [10]
Photoreceptor dysplasia	PD	*PDC*	F-TGTCTATTCCCAGATGAGCATT; R-AAACTCAGCTTCTGGTGCATATC	Zhang, Acland [11]
Progressive rod-cone degeneration	PRCD	*PRCD*	F-TTTCTCCTGCAGACTCTGTCC; R-CAGCTTCTCACGGTTGGA	Zangerl, Goldstein [12]
Autosomal dominant PRA	ADPRA	*RHO*	F-ACACCCGTCTTGTTGGAGAA; R-CCTCAGCAGCACTCTTAGGAC	Kijas, Cideciyan [13]
Progressive retinal atrophy	GR_PRA1	*SLC4A3*	F-CTGCCCAGGTGAGTGCTAGA; R-CACTCCCGTTCAGCTCCAG	Downs, Wallin-Hakansson [14]
Progressive retinal atrophy	GR_PRA2	*TTC8*	F-TGGACTTGGCTGCCCTTT; R-CCATGTCTAAGCCCTTCACAA	Downs, Wallin-Hakansson [15]
Progressive retinal atrophy	PRA	*CNGA1*	F-GCAATCGAAGAACAGCCAAT; R-GCTTTCCCTTCTCTTCTAGCAT	Wiik, Ropstad [16]
		Genotyped individually		
Cone-rod dystrophy	CORD1	*RPGRIP1*	6FAM-GAAGAGCACATGTTGGTGAAGG; R-TGAGCTTTGTTTGCCTTTGG	Mellersh, Boursnell [17], Narfstrom, Jeong [18]

Cone-rod dystrophy (early onset)	EORD	*MAP9*	6FAM-GTCGATGGAGGTCTCCGTAT; R- AACCTGGACCATGGGCAATA	Forman, Hitti [19]
Cone-rod dystrophy	CRD3	*ADAM9*	Nor_F- AAGTACCTACCCTCCCGTTCA; Nor_R- GTAGGATGGAGGTCGAAGAGG; Aff-F- TTGGAACACCTAAATGCCTTG; Aff-R- GTAACCCCAGCCAACACAGTA	Kropatsch, Petrasch-Parwez [20]
Cone-rod dystrophy	CRD	*NPHP4*	F- AGTTCCTTCAGTTACGTCAACCAT; R- CTTCCAACACACAGACAGTACTCC	Wiik, Wade [21]
Rod-cone dystrophy	RCD1	*PDE6B*	F-GAAGAGATCCTGCCCATGTT; R-TGTCCTCTTGTTGCTGCTTCT	Clements, Gregory [22], Suber, Pittler [23]
Rod-cone dystrophy	RCD1a	*PDE6B*	F-GAAGAGATCCTGCCCATGTT; R-TGTCCTCTTGTTGCTGCTTCT	Dekomien, Runte [24]
Rod-cone dystrophy	RCD3	*PDE6A*	F-CAGGACTGGGTGAGGATGATA; R-ACTTGAAATACAGGGCGAGGT	Petersen-Jones, Entz [25]
Rod-cone degeneration	RCD4	*C2ORF71*	6FAM-CCGAGTGCTCCCTCTGTG; R-GGCTGCAGGCCTCGTC	Downs, Bell [26]
Progressive retinal atrophy	PRA3	*FAM161A*	Aff_F- GGATCCCTTTATTTGATTTTAGAAAG; Nor_F- TCCCTTCCTTTTATTTGATTTTAGAAAG; R-6FAM- CAACAAACACAACCTGAGCAA	Downs and Mellersh [27]
Early retinal degeneration	ERD	*STK38L*	F- CAGCCGCACCTAAAATGTAAA; R- CTTTATTCTTGGGTGAACTCCA	Acland, Ray [28], Goldstein, Kukekova [29]

Supplementary Table 2. Multiplex PCR amplification using pooled primers.

Component	Volume/Reaction (μL)	Final Concentration
Primer mix	5	0.2 mM
dNTPs (1.5mM)	2.27	0.2 mM
HotStarTaq Plus 10x PCR buffer	1.70	1x
$MgCl_2$ (25x)	1.02	1.5 mM
HotStarTaq Plus Polymerase (5 units/μL)	0.30	0.09 units/μL
Water	4.71	
Total (μL)	15	

Supplementary Table 3. Thermal cycling conditions for multiplex PCR amplification using pooled primers.

Temperature (°C)	Time	
95	10 min	
95	15 s	25 cycles
60	2 min	
60	30 min	
12	Hold	

Supplementary Table 4. Primer and probe sequences for allelic discrimination assay to determine *NECAP1* genotypes.

Primer	Sequence
qPCR1 primer 1	CAACCAAGAAGGGAGGTGTT
qPCR1 primer 2	GAGGATGGTGGGGAATAGT
qPCR1 probe 1	HEX-CACAGGGGCTGGGGTTTAA-IBFQ
qPCR1 probe 2	FAM-AGACCACAGGGGCTAGGGGTTT-IBFQ

Supplementary Table 5. PCR amplification for the Finnish cohort.

Component	Volume/reaction (µL)	Final Concentration
10× PCR Buffer	1.2	1×
$MgCl_2$ (50mM)	0.4	0.008 mM
dNTP (10mM)	0.24	0.024 mM
Forward primer (5 mM)	1.9	0.38 mM
Reverse primer (5 mM)	1.9	0.38 mM
Betaine (5M)	2	0.4 mM
Biotools DNA Polymerase (5 units/ µL)	0.1	0.02
Water	3.06	
Total (µL)	10.8	0.12

Supplementary Table 6. Primer sequences to amplify SNVs downstream and upstream *NECAP1* variant to determine a disease associated haplotype.

Primer	Sequence
36894608_F	CAGGTGCCCTCAAAGAATCTA
36894608_R	CAGAGCCTTGCTCTAACTCCA
37020314_F	CTTGGAATGCTCCATCTCAAC
37020314_R	CTGCATACTGGTTGGTCGATT
37425357_F	TTCTTGATCTCCAGGGAGAGAG
37425357_R	TCTTCCCTCTGGAAGAGGTTC
37467780_F	TGGAACTTCCCATTCACTGTT
37467780_R	GGTGCTATGCGTTACCACTGT
37501948_F	CCTTCTAGACTCTTGGTAACCACTG
37501948_R	GGGAATACTGGACAGTTACATGC
37541149_F	AACGTAGGCCCAGTGACCTAT
37541149_R	CCACAAGTGCTGGGAGATTTA
37644083_F	AAGATCCAGTCTCTGGCTGGT
37644083_R	CTCTCATTGCCACACAGTCAA
37770618_F	GCCTATAAAGACAGTTCTCCCAAT
37770618_R	GCACCTGCATTTGGTATCATT

Supplementary Table 7. Known breed compositions of 13/15 *NECAP1* heterozygous mixed breed dogs as tested by Wisdom Health, UK.

Dog	Breed composition
**1**	50% Dachshund mix
**2**	50% Pekingese mix, (trace German Spitz)
**3**	25 % Poodle, 12.5% Russell terrier, 12.5 % Vizsla mix (trace German Spitz)
**4**	25% Australian Kelpie, 25% Labrador, mix (trace Dachshund)
**5**	62.5% Dachshund, 12.5% Brittany Spaniel mix
**6**	37.5% American Staffordshire Terrier, 12.5% Labrador, 25% Dutch Shepherd dog mix
**7**	50% German Shepherd dog mix (Trace dachshund)
**8**	50% Labrador mix
**9**	75% Pomeranian mix
**10**	12.5% Rottweiler, 12.5 % Tibetan Mastiff, mix
**11**	25% Poodle, 25% Kritikos Lagonikos, mix (trace Japanese Spitz)
**12**	12.5% Dachshund, 12.5% Pumi, 12.5 % Pekingese mix
**13**	12.5 % Dachshund, 12.5% Rhodesian Ridgeback, 12.5% Kritikos Lagonikos, 12.5% Poodle mix

CanFam3.0 Position	Location from *NECAP1* variant	REF allele	Giant Schnauzer									
			GS_29455_case		GS_32059_case		GS_29746_parent		GS_29747_parent		GS_32603_control	
			Allele 1	Allele 2	Allele 1	Allele 2	Allele 1	Allele 2	Allele 1	Allele 2	Allele 1	Allele 2
27:36894608	573 Kb downstream	G	A	A	A	A	G	A	G	A	G	G
27:37020314	447 Kb downstream	G	A	A	A	A	G	A	G	A	G	G
27:37425357	43 Kb downstream	T	C	C	C	C	T	C	T	C	T	T
27:37467780	4 Kb downstream	G	A	A	A	A	G	A	G	A	G	G
27:37468611	***NECAP1* VARIANT**	**G**	**A**	**A**	**A**	**A**	**G**	**A**	**G**	**A**	**G**	**G**
27:37501948	33 Kb upstream	T	C	C	C	C	T	C	T	C	T	T
27:37541149	72 Kb upstream	G	A	A	A	A	G	A	G	A	G	G
27:37644083	175 Kb upstream	A	G	G	G	G	A	G	A	G	A	G
27:37770618	301 Kb upstream	A	G	G	G	G	A	G	A	G	A	G

CanFam3.0 Position	Giant Spitz		MLHD													
	Giant_Spitz104		MLHD 27322		MLHD_7094		MLHD_6445		MLHD_5741		MLHD_6441		MLHD_5128		MLHD_1617	
	Allele 1	Allele 2	Allele 1	Allele 2	Allele 1	Allele 2	Allele 1	Allele 2	Allele 1	Allele 2	Allele 1	Allele 2	Allele 1	Allele 2	Allele 1	Allele 2
27:36894608	G	G	G	G	A	A	G	A	G	G	A	A	G	G	G	G
27:37020314	G	A	G	G	G	G	G	A	G	G	G	G	G	G	G	G
27:37425357	T	C	T	C	T	C	T	C	T	C	T	C	T	C	T	T
27:37467780	G	A	G	A	G	A	G	A	G	A	G	A	G	A	G	G
27:37468611	**G**	**A**	**G**	**A**	**G**	**A**	**G**	**A**	**G**	**A**	**G**	**A**	**G**	**A**	**G**	**G**
27:37501948	T	C	T	C	T	C	T	C	T	C	T	C	T	C	T	T
27:37541149	G	A	G	A	G	A	G	A	G	A	G	A	G	A	G	G
27:37644083	A	G	A	A	A	A	-	-	A	A	A	A	A	A	A	A
27:37770618	G	G	G	G	G	G	A	G	A	G	A	G	A	G	A	A

Figure S1. To build a disease-associated haplotype, a total of eight SNVs up to 301 kb upstream and 573 kb downstream of the *NECAP1* variant were genotyped in two PRA affected GS *NECAP1* homozygotes, both *NECAP1*$^{+/-}$ unaffected parents, and an unaffected *NECAP1*$^{+/+}$ GS; one *NECAP1*$^{+/-}$ Giant Spitz from the DBVDC; six *NECAP1*$^{+/-}$ Miniature Longhaired Dachshunds and one *NECAP1*$^{+/+}$ Miniature Longhaired Dachshund. A disease associated haplotype (shaded orange) was determined. The alternate allele is shaded yellow.

References

1. Sidjanin, D.J.; Lowe, J.K.; McElwee, J.L.; Milne, B.S.; Phippen, T.M.; Sargan, D.R.; Aguirre, G.D.; Acland, G.M.; Ostrander, E.A. Canine CNGB3 mutations establish cone degeneration as orthologous to the human achromatopsia locus ACHM3. *Hum. Mol. Genetic.* **2002**, *11*, 1823–1833.
2. Yeh, C.Y.; Goldstein, O.; Kukekova, A.V.; Holley, D.; Knollinger, A.M.; Huson, H.J.; Pearce-Kelling, S.E.; Acland, G.M.; Komaromy, A.M. Genomic deletion of CNGB3 is identical by descent in multiple canine breeds and causes achromatopsia. *BMC Genet*. **2013**, *14*, 27.
3. Tanaka, N.; Dutrow, E.V.; Miyadera, K.; Delemotte, L.; MacDermaid, C.M.; Reinstein, S.L.; Crumley, W.R.; Dixon, C.J.; Casal, M.L.; Klein, M.L.; et al. Canine CNGA3 Gene Mutations Provide Novel Insights into Human Achromatopsia-Associated Channelopathies and Treatment. *PloS ONE* **2015**, *10*, e0138943.
4. Aguirre, G.D.; Baldwin, V.; Pearce-Kelling, S.; Narfstrom, K.; Ray, K.; Acland, G.M. Congenital stationary night blindness in the dog: common mutation in the RPE65 gene indicates founder effect. *Mol. Vis.* **1998**, *4*, 23.
5. Veske, A.; Nilsson, S.E.; Narfstrom, K.; Gal, A. Retinal dystrophy of Swedish briard/briard-beagle dogs is due to a 4-bp deletion in RPE65. *Genomics* **1999**, *57*, 57–61.
6. Guziewicz, K.E.; Zangerl, B.; Lindauer, S.J.; Mullins, R.F.; Sandmeyer, L.S.; Grahn, B.H.; Stone, E.M.; Acland, G.M.; Aguirre, G.D. Bestrophin gene mutations cause canine multifocal retinopathy: a novel animal model for best disease. *Invest. Ophthalmol. Vis. Sci.* **2007**, *48*, 1959–1967.
7. Zangerl, B.; Wickstrom, K.; Slavik, J.; Lindauer, S.J.; Ahonen, S.; Schelling, C.; Lohi, H.; Guziewicz, K.E.; Aguirre, G.D. Assessment of canine BEST1 variations identifies new mutations and establishes an independent bestrophinopathy model (cmr3). *Mol. Vis.* **2010**, *16*, 2791–2804.
8. Lippmann, T.; Jonkisz, A.; Dobosz, T.; Petrasch-Parwez, E.; Epplen, J.T.; Dekomien, G. Haplotype-defined linkage region for gPRA in Schapendoes dogs. *Mol. Vis*. **2007**, *13*, 174–180.
9. Dekomien, G.; Vollrath, C.; Petrasch-Parwez, E.; Boeve, M.H.; Akkad, D.A.; Gerding, W.M.; Epplen, J.T. Progressive retinal atrophy in Schapendoes dogs: mutation of the newly identified CCDC66 gene. *Neurogenetics* **2010**, *11*, 163–174.
10. Ahonen, S.J.; Arumilli, M.; Lohi, H. A CNGB1 frameshift mutation in Papillon and Phalene dogs with progressive retinal atrophy. *PloS ONE* **2013**, *8*, e72122.
11. Zhang, Q.; Acland, G.M.; Parshall, C.J.; Haskell, J.; Ray, K.; Aguirre, G.D. Characterization of canine photoreceptor phosducin cDNA and identification of a sequence variant in dogs with photoreceptor dysplasia. *Gene.* **1998**, *215*, 231–239.
12. Zangerl, B.; Goldstein, O.; Philp, A.R.; Lindauer, S.J.; Pearce-Kelling, S.E.; Mullins, R.F.; Graphodatsky, A.S.; Ripoll, D.; Felix, J.S.; Stone, E.M.; et al. Identical mutation in a novel retinal gene causes progressive rod-cone degeneration in dogs and retinitis pigmentosa in humans. *Genomics* **2006**, *88*, 551–563.
13. Kijas, J.W.; Cideciyan, A.V.; Aleman, T.S.; Pianta, M.J.; Pearce-Kelling, S.E.; Miller, B.J.; Jacobson, S.G.; Aquirre, G.D.; Acland, G.M. Naturally occurring rhodopsin mutation in the dog causes retinal dysfunction and degeneration mimicking human dominant retinitis pigmentosa. *Proc. Natl. Acad. Sci. USA* **2002**, *99*, 6328–6333.
14. Downs, L.M.; Wallin-Hakansson, B.; Boursnell, M.; Marklund, S.; Hedhammar, A.; Truve, K.; Hübinette, L.; Lindblad-Toh, K.; Bergström, T.; Mellersh, C.S. A frameshift mutation in golden retriever dogs with progressive retinal atrophy endorses SLC4A3 as a candidate gene for human retinal degenerations. *PloS ONE* **2011**, *6*, e21452.
15. Downs, L.M.; Wallin-Hakansson, B.; Bergstrom, T.; Mellersh, C.S. A novel mutation in TTC8 is associated with progressive retinal atrophy in the golden retriever. *Canine Genet. Epidemiol.* **2014**, *1*, 4.
16. Wiik, A.C.; Ropstad, E.O.; Ekesten, B.; Karlstam, L.; Wade, C.M.; Lingaas, F. Progressive retinal atrophy in Shetland sheepdog is associated with a mutation in the CNGA1 gene. *Anim. Genetic.* **2015**, *46*, 515–521.
17. Mellersh, C.S.; Boursnell, M.E.; Pettitt, L.; Ryder, E.J.; Holmes, N.G.; Grafham, D.; Forman, O.P.; Sampson, J.; Barnett, K.C.; Blanton, S.; et al. Canine RPGRIP1 mutation establishes cone-rod dystrophy in miniature longhaired dachshunds as a homologue of human Leber congenital amaurosis. *Genomics* **2006**, *88*, 293–301.
18. Narfstrom, K.; Jeong, M.; Hyman, J.; Madsen, R.W.; Bergstrom, T.F. Assessment of hereditary retinal degeneration in the English springer spaniel dog and disease relationship to an RPGRIP1 mutation. *Stem Cells Internat*. **2012**, *2012*, 685901.
19. Forman, O.P.; Hitti, R.J.; Boursnell, M.; Miyadera, K.; Sargan, D.; Mellersh, C. Canine genome assembly correction facilitates identification of a MAP9 deletion as a potential age of onset modifier for RPGRIP1-associated canine retinal degeneration. *Mamm Genome.* **2016**, *27*, 237–245.

20. Kropatsch, R.; Petrasch-Parwez, E.; Seelow, D.; Schlichting, A.; Gerding, W.M.; Akkad, D.A.; Epplen, J.T.; Dekomien, G. Generalized progressive retinal atrophy in the Irish Glen of Imaal Terrier is associated with a deletion in the ADAM9 gene. *Mol. Cell Probes* **2010**, *24*, 357–363.
21. Wiik, A.C.; Wade, C.; Biagi, T.; Ropstad, E.O.; Bjerkas, E.; Lindblad-Toh, K.; et al. A deletion in nephronophthisis 4 (NPHP4) is associated with recessive cone-rod dystrophy in standard wire-haired dachshund. *Genome Res.* **2008**, *18*, 1415–1421.
22. Clements, P.J.; Gregory, C.Y.; Peterson-Jones, S.M.; Sargan, D.R.; Bhattacharya, S.S. Confirmation of the rod cGMP phosphodiesterase beta subunit (PDE beta) nonsense mutation in affected rcd-1 Irish setters in the UK and development of a diagnostic test. *Curr. Eye Res.* **1993**, *12*, 861–866.
23. Suber, M.L.; Pittler, S.J.; Qin, N.; Wright, G.C.; Holcombe, V.; Lee, R.H.; Craft, C.M.; Lolley, R.N.; Baehr, W.; Hurwitz, R.L. Irish setter dogs affected with rod/cone dysplasia contain a nonsense mutation in the rod cGMP phosphodiesterase beta-subunit gene. *Proc. Natl. Acad. Sci. USA* **1993**, *90*, 3968–3972.
24. Dekomien, G.; Runte, M.; Godde, R.; Epplen, J.T. Generalized progressive retinal atrophy of Sloughi dogs is due to an 8-bp insertion in exon 21 of the PDE6B gene. *Cytogenet. Cell Genetic.* **2000**, *90*, 261–267.
25. Petersen-Jones, S.M.; Entz, D.D.; Sargan, D.R. cGMP phosphodiesterase-alpha mutation causes progressive retinal atrophy in the Cardigan Welsh corgi dog. *Invest. Ophthalmol. Vis. Sci.* **1999**, *40*, 1637–1644.
26. Downs, L.M.; Bell, J.S.; Freeman, J.; Hartley, C.; Hayward, L.J.; Mellersh, C.S. Late-onset progressive retinal atrophy in the Gordon and Irish Setter breeds is associated with a frameshift mutation in C2orf71. *Anim. Genetic.* **2013**, *44*, 169–177.
27. Downs, L.M.; Mellersh, C.S. An Intronic SINE insertion in FAM161A that causes exon-skipping is associated with progressive retinal atrophy in Tibetan Spaniels and Tibetan Terriers. *PloS ONE* **2014**, *9*, e93990.
28. Acland, G.M.; Ray, K.; Mellersh, C.S.; Langston, A.A.; Rine, J.; Ostrander, E.A.; Aguirre, G.D. A novel retinal degeneration locus identified by linkage and comparative mapping of canine early retinal degeneration. *Genomics* **1999**, *59*, 134–142.
29. Goldstein, O.; Kukekova, A.V.; Aguirre, G.D.; Acland, G.M. Exonic SINE insertion in STK38L causes canine early retinal degeneration (erd). *Genomics* **2010**, *96*, 362–368.
